# Bilateral Fetal Hydrothorax Requiring Intrauterine Fetal Thoracoamniotic Shunts: Anesthetic Considerations and Management

**DOI:** 10.1100/tsw.2009.64

**Published:** 2009-06-12

**Authors:** John J. Hache, Stephen P. Emery, Manuel C. Vallejo

**Affiliations:** ^1^ Department of Anesthesiology, Fetal Diagnosis and Treatment Center, Magee Women's Hospital, University of Pittsburgh School of Medicine, Pittsburgh, PA, USA; ^2^ Department of Obstetrics and Gynecology, Fetal Diagnosis and Treatment Center, Magee Women's Hospital, University of Pittsburgh School of Medicine, Pittsburgh, PA, USA

**Keywords:** anesthesiology, obstetrics, fetal surgery, hydrops fetalis, intravenous sedation

## Abstract

After prenatal diagnosis of bilateral fetal hydrothorax, ascites, and polyhydramnios, bilateral thoracoamniotic shunts were placed at 29 weeks gestation using an ultrasound-guided, minimally invasive technique. Anesthetic care was managed using intravenous sedation and local anesthesia infiltration. The anesthetic considerations for such procedures are discussed.

## INTRODUCTION

Bilateral fetal hydrothorax with resultant hydrops fetalis is a life-threatening malformation, but is potentially correctable *in utero* with minimally invasive fetal surgery[1]. In this report, we present the management and anesthetic challenges when caring for a mother undergoing ultrasound-guided percutaneous placement of bilateral fetal thoracoamniotic shunts, while receiving intravenous sedation and local anesthesia infiltration.

## CASE REPORT

A 29-year-old, gravida-3, para-1, 95-kg, 160-cm, woman was diagnosed with bilateral fetal hydrothorax at 21 weeks gestation. At that time, normal fetal cardiac anatomy and function were noted, with no identified cardiac etiology for the effusions. Other causes of secondary hydrothorax were excluded by detailed sonographic assessment and maternal history. Fetal thoracentesis was performed at 27 weeks gestation under local anesthesia and was well tolerated by the mother, but fluid reaccumulated within 1 week. At that time, increasing bilateral hydrothorax, ascites, polyhydramnios, and concern for progression to hydrops fetalis were present. If allowed to continue without treatment, fetal death would be imminent[2]. Delivery was not an optimal option due to early gestation and developing hydrops, the combination of which renders a poor neonatal prognosis. A decision was made to perform ultrasound-guided percutaneous placement of bilateral fetal thoracoamniotic shunts.

The patient had a history of hypercholesterolemia and had undergone a past cholecystectomy. Her current medications included only prenatal vitamins. Physical examination revealed no significant abnormalities, a Mallampati class 1 airway, regular heart tones without murmur, and clear lung fields. There was no sign of maternal edema or neurologic deficit. Initial laboratory results revealed values within normal limits. Amniocentesis performed at 15 weeks gestation demonstrated a normal karyotype, and negative toxoplasmosis, syphilis, rubella, cytomegalovirus, human immunodeficiency virus, and herpes simplex virus studies were obtained on maternal blood and amniotic fluid. Two 12-mg doses of intramuscular betamethasone were administered over 2 days prior to shunt placement for promotion of fetal lung maturity. Indomethacin, 50 mg orally, was given preoperatively for tocolysis[3]. Sodium bicitrate, 30 ml orally, was also administered preoperatively for aspiration prophylaxis. Subsequently, the patient was taken to the operating suite for ultrasound-guided percutaneous placement of bilateral fetal thoracoamniotic shunts.

At 29 weeks and 6 days of gestation, the procedure was conducted in a standard obstetric operating room, with equipment and personnel immediately available for emergent Caesarean delivery in the event of nonreassuring fetal status[4]. Anesthetic management included preparation for emergent rapid sequence induction of general anesthesia should the situation warrant a Caesarean section.

The patient was placed on the operating room table in the supine position with left uterine displacement; oxygen via facemask at 8 l/min was provided. Standard maternal monitors including noninvasive blood pressure, continuous electrocardiogram heart rate and rhythm monitoring, and continuous pulse-oximetry were applied in addition to capnography and intermittent skin temperature monitoring[5]. Initial vital signs revealed a blood pressure of 132/73 mmHg, respiratory rate of 17, and a heart rate of 106 beats per minute (bpm). The patient was administered small intermittent bolus doses of midazolam, fentanyl, and propofol for intravenous sedation. The procedure was conducted using a 3-mm trocar and cannula passed, under ultrasound guidance, through the maternal abdomen, uterus, amniotic cavity, and directly into the fetal left chest base. The goal is for fetal chest fluid to drain into the amniotic space, allowing fetal lung expansion. Prior to initiating placement of trocars, 1% lidocaine solution was infiltrated into the skin and subcutaneous tissues by the surgeon[6,7]. With placement and manipulation of trocars, propofol boluses were titrated to patient comfort. Midazolam and fentanyl alone provided an inadequate level of comfort that became adequate with the addition of propofol. External fetal heart rate monitoring was initiated preoperatively and subsequently replaced with continuous ultrasound monitoring. The initial ultrasound images (Fig. 1) demonstrate fetal lung collapse and hydrothorax. An 8 French Cook pigtail catheter was deployed into the fetal thoracic cavity. Free flow of chest fluid was identified through the cannula, confirming proper position. A similar procedure was performed on the right using a second percutaneous puncture site.

Prior to terminating the procedure, excessive amniotic fluid was removed until the amniotic fluid volume was judged grossly normal on ultrasound by the surgeon. Minimal surgical blood loss was noted; estimated to be less than 5 cc. The patient remained awake, alert, and cooperative throughout the entire procedure, without complaint or significant movement. There were no significant deviations in heart rate, blood pressure, or respiratory rate throughout the procedure. The entire procedure lasted 47 min. A total dose of 4 mg midazolam and 250 μg fentanyl was required. These medications were titrated to the patient's level of comfort and respiratory rate over 10 min prior to the initial skin puncture and redosed every 15 min as needed. Intermittent boluses of propofol were also infused in 10-mg increments and titrated to the level of surgical stimulation. A total dose of 140 mg of propofol was administered. Propofol was titrated in 10-mg increments every 5–10 min, with careful titration of 80 mg over approximately 15–20 min when more forceful manipulation of the trocars was required. The patient was able to respond to gentle verbal commands throughout the procedure.

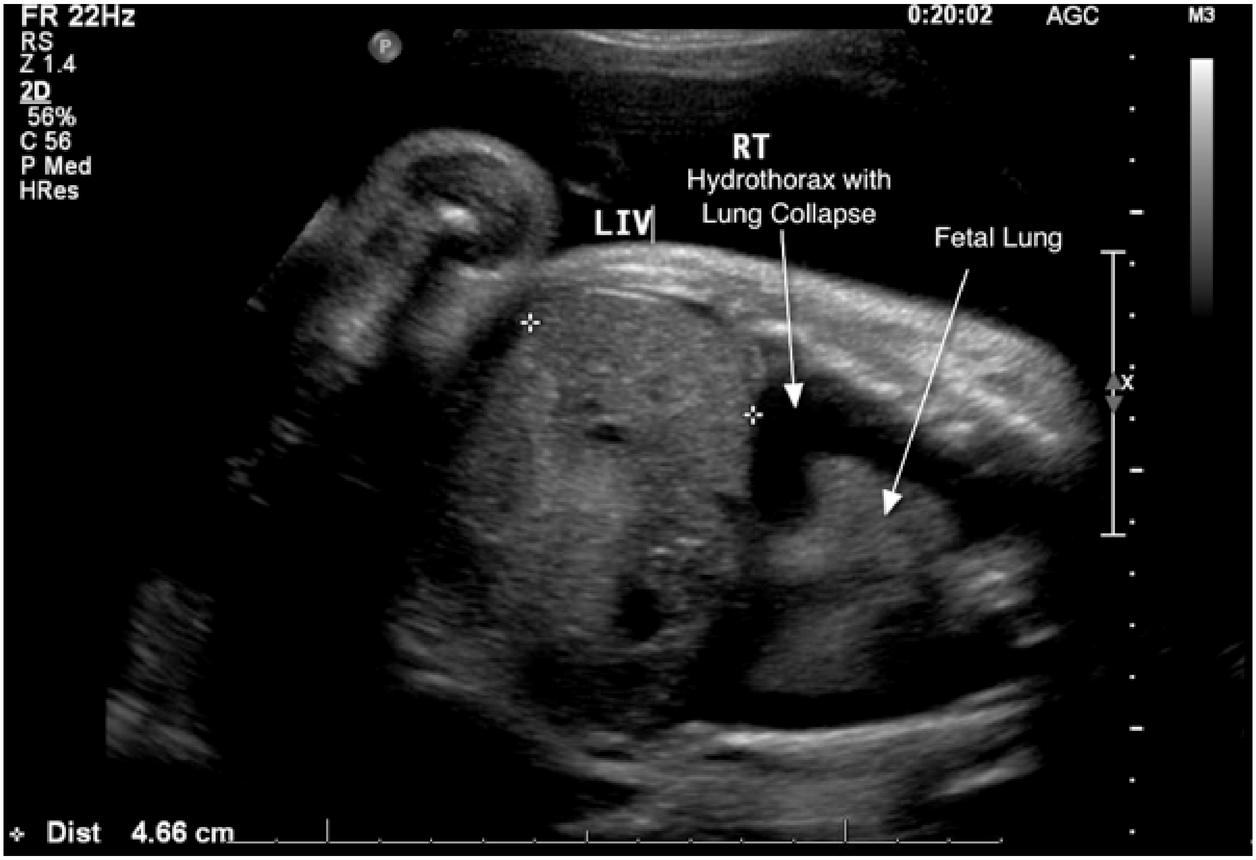

**FIGURE 1.** Ultrasound image at 29 weeks and 6 days gestation demonstrating fetal hydrothorax (left arrow) resulting in collapse of the fetal lungs (right arrow). The liver (LIV) is well visualized. The right side of the fetus is marked RT and the fetal head lies to the right of the ultrasound image.

The patient recovered in the labor and delivery suite with monitoring of fetal heart rate and uterine contractions for 4 h, the standard time for postoperative monitoring at our institution for these procedures. On discharge from the labor and delivery suite, the fetal heart rate was reassuring and at the normal baseline rate (140–150 bpm) prior to the surgical procedure. For mild postprocedure uterine contractions, nifedipine 20 mg was given by mouth every 6 h until hospital discharge, at which time contractions had ceased. She was discharged to home the next day, with close outpatient follow-up.

The follow-up interview with the patient prior to discharge revealed that the patient did not recall significant distress during the procedure, although she was able to recount operating room events. The follow-up ultrasound imaging revealed improved fetal status. At approximately 32 weeks gestation, there was no evidence of hydrops fetalis, the amniotic fluid index was normal at 23.1 cm, and fetal ascites had resolved. Near complete expansion of the lungs was noted (Fig. 2) and antenatal testing remained reassuring. Further evaluation included serial ultrasound every 2 weeks for the duration of pregnancy.

The patient delivered at full term via spontaneous vaginal delivery. The neonate did not require endotracheal intubation or ventilatory support, and required no intervention other than removal of the thoracoamniotic shunts. The newborn was evaluated by the neonatology service and subsequently discharged from their care.

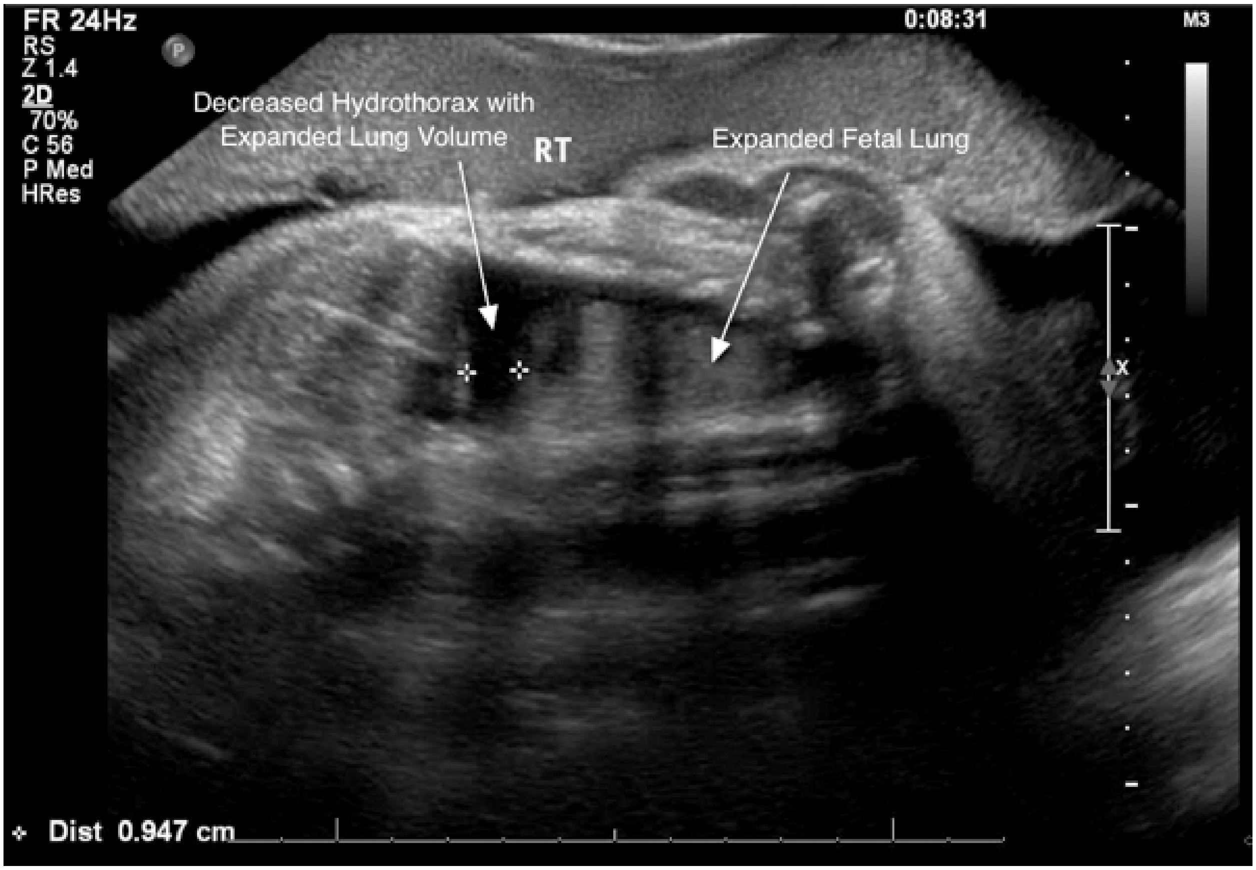

**FIGURE 2.** Follow-up fetal ultrasound at 32 weeks. Hydrops fetalis has resolved and expansion of the fetal lung can be seen (right arrow). Residual fetal hydrothorax is decreased in size (left arrow).

## DISCUSSION

Fetal surgery is indicated when the fetal risk of no intervention outweighs surgical risks and the maternal procedural risk is low[2]. Contraindications for fetal surgery include lethal genetic diseases in the fetus or other malformations that would limit the effective outcome of the planned intervention. Serious maternal diseases, such as preeclampsia, also contraindicate fetal surgery[7]. Bilateral fetal hydrothorax is amenable to minimally invasive fetal surgery because the malformation is life threatening, potentially correctable, and the risks to the mother are low. The safety profile is increased when using a minimally invasive approach, whereby hysterotomy is avoided. Additional benefits of a minimally invasive approach include the potential for a future vaginal delivery and minimal obstetric anesthetic requirements.

The anesthetic goals for both the mother and fetus include maintaining hemodynamic stability, maternal oxygenation, normocarbia, placental perfusion, uterine relaxation, and preparation for a potential Caesarean section. This was accomplished with careful titration of sedative medications and adequate local anesthetic use by the surgeon. The safety of this anesthetic approach has been demonstrated for percutaneous laser treatment of twin-twin transfusion syndrome[6]. Throughout the procedure, close communication with the patient and surgeon allowed for the patient to communicate her level of discomfort and anxiety, with the surgeon proceeding only after the proper level of patient comfort had been achieved. High levels of vigilance are required when administering propofol either by infusion or bolus dose titration. Before and after each dose of propofol, the patient's mental status and vital signs were carefully reviewed, with attention given to the cumulative effects of subsequent doses.

The decision to avoid epidural and spinal anesthesia was made in order to avoid sympathectomy and its hemodynamic consequences, given the minimally invasive nature of the procedure that required small skin, uterine, and fetal incisions with low levels of expected pain and uterine activity. Furthermore, while neuraxial anesthetic techniques may provide dense analgesia, they do not provide anxiolysis or uterine relaxation by themselves.

Intravenous sedation and local anesthetic infiltration was acceptable for this patient, primarily because of the patient's Mallampati class I airway and lack of confounding conditions (i.e., morbid obesity), and the patient was highly motivated and cooperative. Adequate time for infiltration of local anesthetics to take effect and open communication with a cooperative surgeon are important factors in the success of the procedure. If immediate delivery was necessary, emergent rapid sequence induction of general anesthesia with cricoid pressure was planned. Because of the short operative time, expected to be approximately 30–45 min, and in an effort to limit total doses and fetal exposure to intravenous medications, we opted to titrate intravenous medication instead of using a continuous intravenous infusion.

The disadvantages of using intravenous medications include profound respiratory depression with resultant life-threatening airway compromise. Difficult mask ventilation and endotracheal intubation are common concerns to parturients who are particularly at risk for aspiration. The importance of small, carefully titrated doses of propofol with vigilant attention to vital signs, patient status, and degree of sedation must be emphasized, and is especially important with regard to maintaining fetal status and limiting fetal exposure to intravenous medications.

There has been concern regarding harmful and teratogenic effects of anesthetic agents on the fetus. However, this is usually a consideration limited to the first trimester. Benzodiazepines, once thought to be teratogenic, are now considered to be safe[8]. However, benzodiazepines are listed as a Category D classification for use in pregnancy by the Food and Drug Administration (FDA). Propofol in the fetus induces decreased fetal activity and, hence, causes decreased fetal heart rate variability. With the use of ultrasonography, fetal activity was continually monitored and assessed throughout the procedure[9].

The surgeon communicated concerns that, on traversing the uterus, contractions could lead to placental hypoperfusion and fetal distress necessitating delivery. In this scenario, delivery of the fetus can be difficult without uterine relaxation. In the event that uterine contractions became significant, uterine relaxation was to be provided with nitroglycerin, 60-μg intravenous doses titrated to effect every 2–3 min[10]. Small doses of nitroglycerine are frequently administered in conjunction with neuraxial anesthesia without producing significant or persistent hypotension. If profound uterine relaxation became necessary, general anesthesia was planned.

Fetal monitoring was conducted using ultrasonography during the procedure, and fetal status and movement could be easily assessed. Fetal tachycardia or excessive fetal movement during the procedure may have signified inadequate fetal anesthesia. In general, fetal paralysis is indicated when fetal movement is anticipated to be a problem. This was not an issue in this particular case, as the fetus was moribund. Fetal paralysis requires an additional fetal puncture, carrying additional risks to both the mother and fetus. Our anesthetic technique involved agents that readily cross the placenta, and the absence of fetal tachycardia or gross fetal movement suggested that fetal stimulation was minimal[8]. Fetal movements for thoracoamniotic shunt placement could create problems when trying to place the shunts properly through the fetal chest wall. If fetal movements had become excessive, the surgeon had the option of providing a fetal intramuscular injection of fentanyl and/or a neuromuscular blocking agent to improve operative conditions. At the termination of the procedure, external fetal heart rate monitoring was used to continue to monitor the fetus.

In conclusion, the anesthetic management and treatment of a parturient with a bilateral fetal hydrothorax, requiring intrauterine fetal thoracoamniotic shunts under intravenous sedation and local anesthesia infiltration, is presented. Common pitfalls and obstacles to the safe administration of intravenous sedation to parturients, and concerns regarding premature labor and fetal status are discussed.
